# Child marriage and risky health behaviors: an analysis of tobacco use among early adult and early middle-aged women in India

**DOI:** 10.1186/s12905-022-01781-3

**Published:** 2022-06-03

**Authors:** Biplab K. Datta, Ashwini Tiwari, Ishtiaque Fazlul

**Affiliations:** 1 Institute of Public and Preventive Health, Augusta University, 1120 15th Street, Augusta, GA USA; 2 Department of Population Health Sciences, Medical College of Georgia, Augusta University, Augusta, GA USA; 3 Department of Economics, University of Missouri, Columbia, MO USA; 4 Present Address: Coles College of Business, Kennesaw State University, Kennesaw, GA USA

**Keywords:** Child marriage, Tobacco, Risky health behavior, Life course, India

## Abstract

**Background:**

Though the harmful impacts of child marriage have been evaluated across several domains, evidence on the relationship between child marriage and health behaviors over the life course is limited. In this paper, we examined whether getting married as a child is associated with one of the most common risky health behaviors, tobacco use, in adulthood.

**Methods:**

Using nationally representative data from India, we compared the odds in favor of tobacco use among early adult (age 22–34) and early middle-aged (age 35–44) women who were married before age 18 with that of those who were married as youths (age 18–21). We estimated univariate and multivariable logistic regressions to obtain odds ratios in favor of any tobacco-use and relative risk ratios in favor of mutually exclusive types of tobacco use (smoking-only, smokeless-only, and dual-use). We also explored the intensive margin of the relationship by assessing if the odds of tobacco use in adulthood were affected by how early (13 or less, 14–15, or 16–17) a child bride was married.

**Results:**

We find that the adjusted odds of tobacco use for those who were married as a child were 1.3 and 1.2 times that of those who were married as a youth among early adult and early middle-aged women, respectively. The younger was the child bride when married, the higher were the odds of tobacco use as an adult. The relative risks of different types of tobacco use were also higher for child brides than their peers.

**Conclusions:**

These results are the first evidence of the association between child marriage and a major risky health behavior, tobacco use, over the life course. These findings will inform policies to strengthen child marriage prevention efforts and targeted tobacco control initiatives in the low-and-middle income countries.

**Supplementary Information:**

The online version contains supplementary material available at 10.1186/s12905-022-01781-3.

## Background

Child marriage, defined as a union occurred before age 18 years, is considered a health and human right violation [1]. The detrimental impacts of girl child marriage have been demonstrated across several domains including child and maternal health, intimate partner violence, educational attainment, and economic wellbeing [2]. Further, child marriage hinders decision making and bargaining power [2–4], limits access to social support [5], and hinders the child bride’s ability to negotiate sexual activity, contraceptive use and birth-spacing [1, 6]. Child marriage may lead to isolation and depressions as well as malnutrition and adverse reproductive outcomes [7, 8]. Together, these findings reiterate the threat towards a child bride’s physical and mental wellbeing.

While child marriage is not readily described as an adverse childhood experience (ACE) in the literature to date, it is a recognized form of violence against children and adolescents [9]. Like child marriage, ACE entails exposures to psychological, physical, and sexual forms of abuse as well as household dysfunction including domestic violence [10]. As such, it is plausible that child marriage, as a chronic and potentially traumatic exposure, may lead to behavioral problems in adulthood as seen with the ACEs. For example, a well-established body of literature highlights associations between the ACEs and risky health behaviors in adulthood such as sexual risk taking, obesity, and excessive alcohol use [10–12]. However, not many studies explore the relationship between child marriage and such outcomes over the life course. A couple of recent studies find that child marriage increases the risk of chronic conditions among women at youth and middle age [13, 14].

In low-and-middle income countries (LMICs), where child marriage is most prevalent, other ACE-associated health behaviors, such as tobacco use, are a rising concern. Almost 80% of the 1.3 billion tobacco users in the world live in the LMICs, which bear a disproportionately high burden of tobacco related illness and death [15]. India, for example, a lower-middle income country, accounts for 9% of the world’s tobacco users with more than 115 million adults consuming some kind (smoking, smokeless, or both) of tobacco products [16]. Tobacco-use in India takes a toll of more than 1 million tobacco-related deaths annually [17], along with an estimated annual economic cost of approximately 1% of the GDP [18]. While childhood adversity such as physical, verbal, and sexual abuse are associated with tobacco use behaviors [12, 19], evidence on the relationship of child marriage with tobacco use is limited in existing literature.

Many social-structural and psychosocial factors including gender roles and responsibilities, low socioeconomic status, early motherhood as well as sexual abuse and interpersonal violence influence tobacco use in women [20, 21]. Tobacco-use is also regraded as a tool of socialization and facilitator of social relationship among women [22]. Further, tobacco-use initiation can be influenced by family members’ tobacco consumption and misconceptions about its health impacts [22]. Tobacco-use can also be impacted by spousal tobacco-use behavior [23] and early sexual initiation [24]. Many of these factors are pertinent to child brides, who lack voice and agency within the household and are forced to perform adult roles before reaching adulthood. Furthermore, child brides suffer from stress and anxiety [25], which can promote tobacco use in women [26]. All this evidence indicates a likely relationship between child marriage and tobacco use.

The goal of this study is to examine the association between tobacco consumption and child marriage in India, one of the most populous countries and home of the highest number of child brides worldwide [9]. Specifically, we investigated whether getting married as a child is associated with female tobacco use in early adulthood and early middle-age, defined as ages 22 to 34 and 35 to 44, respectively [27]. The analyses serve as an assessment of the relationship between girl child marriage and health-harming behaviors over the life course and provide evidence on how adverse circumstances analogous to ACEs are related to risky health behaviors in the developing country context. The findings, therefore, inform policies to strengthen child marriage prevention efforts and tobacco control initiatives in the LMICs.

## Methods

### Data

**Fig. 1:**
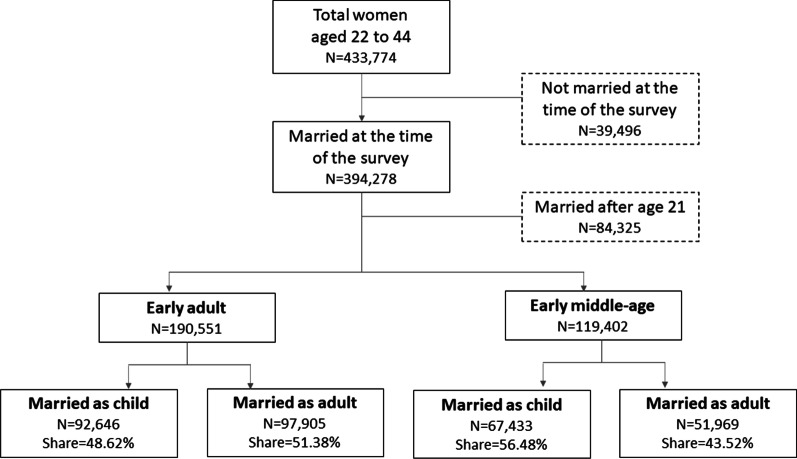
Flow diagram of study participants

We used data from the India National Family Health Survey (NFHS) 2015-16. The NFHS is a nationally representative survey that collects sociodemographic and anthropometric information of women aged 15 to 49 years covering urban and rural areas across all states and union territories of India [28]. For our analysis, we obtained information of 309,953 women aged 22 to 44 years, who were married by age 21. We confined the sample to those who were married by age 21 for two reasons—first, since our study population is of age 22 to 44, confining marriage age prior to age 22 ensures uniformity of the study sample; and second, those who were married during youth or late adolescence (between age 18 to 21 years) were more comparable to those who were married as child (before age 18 years). Our results, however, were not dependent on this restriction and the results hold when no marital age restrictions were imposed. Among the study participants, 61.5% were in their early adulthood and 38.5% were in early middle age. Figure 1 presents the flow diagram of study participants.

### Measures

The NFHS reports respondent’s age at first marriage, from which we identified women who were married as child (by age 17) and as youth (between age 18 to 21). The NFHS also provides information on self-reported consumption of smoking tobacco products such as cigarette, bidi, cigar, pipe, hookah; and smokeless tobacco products such as paan masala or gutkha with tobacco, khaini, paan with tobacco, chewing tobacco, snuff, and other tobacco products. Respondents were asked whether they currently (at the time of the survey) smoke or use tobacco in any other form. Respondents answering this question in the affirmative were recorded as tobacco users.

### Statistical analysis

We first compared the tobacco-use prevalence of women at early adulthood and early middle age by child marriage and by age at first marriage as follows: 13 or less, 14 to 15, 16 to 17, and 18 to 21. Next, we estimated univariate and multivariable binomial logistic regressions to obtain unadjusted and adjusted odds ratios in favor of tobacco use. Our dependent variable is a binary variable indicating whether the respondent consumes any tobacco products (smoking, smokeless, or both) or not. Our main explanatory variable is another binary variable indicating whether the respondent was married as a child or as a youth—taking the value 1 if married by age 17, and 0 if married between age 18 to 21.

In the multivariable specification, we controlled for a rich set of covariates including respondent’s age (as continuous variable); educational attainment—no education (reference category), primary, secondary, and higher; household size—3 or less (reference category), 4 to 5, 6 to 8, and 9 or more; household wealth index quintiles—poorest (reference category), poorer, middle, richer, and richest; religion—Hindu (reference category), Muslim, Christian, Sikh, Buddhist, and other; caste – not socially or economically backward class (reference category), scheduled caste, scheduled tribe, and other backward class; urban or rural residence; and state fixed effects. This set of covariates is common in both child marriage [29, 30] and tobacco use [31, 32] literature.

We then examined how child marriage is associated with mutually exclusive tobacco-use types—smoking only, smokeless only, and dual-use (both smoking and smokeless) for the early adult and early middle-age groups. We estimated a multinomial logistic regression model to obtain relative risk ratios (RRR) in favor of types of tobacco use compared to the base outcome of “no tobacco use”. Like the binomial logistic model, we estimated the multinomial model with and without other control variables.

Next, to evaluate if child marriage at a younger age has a different relationship with tobacco consumption compared to child marriage at an older age, we estimated another binomial logistic model examining the odds in favor of tobacco use for child marriage at different ages—13 or less, 14 to 15, and 16 to 17. We regressed the tobacco-use indicator variable on a set of binary variables indicating each of the three child marriage age categories. Age at youth (18 to 21) serves as the reference category under this specification. We estimated the model with and without other covariates of the previous models. Next, to check the robustness of our results, we estimated the original bivariate and multivariate specifications for four sub-groups of women by wealth and geographic location—urban poor, urban non-poor, rural poor, and rural non-poor; and two additional sub-groups of women by educational attainment—low (primary or no education) and high (secondary or higher). Poor is defined as being at the bottom-two quintiles of the household wealth index distribution. Since there exist a sub-national heterogeneity in child marriage prevalence in India [33], for further robustness check, we estimated the model for the sub-groups of 28 administrative states of India (results presented in Additional file 1: Appendix). All models were estimated for the full sample (age 22 to 44 years), and sub-samples of early adult (age 22 to 34 years) and early middle-age (age 35 to 44 years) using complex survey weights.

## Results

In our sample, around 50% of the women at early adulthood and nearly 60% of the women at early middle-age were married as children (before age 18). Table 1 presents the background characteristics of respondents in both age groups (early adult and early middle-aged) by child marriage. Across both groups, those who were married as a child were more likely to have lower educational attainment. Prevalence of child marriage was higher in rural areas and lower among those who belonged to wealthier households.

**Table 1:** Background characteristics of early adult and early middle-aged women by child marriage

	Early adulthood (22–34)		Early middle age (35–44)		All (22–44)	
	No child marriage	Child marriage	No child marriage	Child marriage	No child marriage	Child marriage
*Share (%)*						
Tobacco use						
Any tobacco	4.62	7.88	9.42	12.63	6.26	9.89
	(4.44, 4.80)	(7.62, 8.14)	(9.05, 9.79)	(12.26, 13.00)	(6.08, 6.44)	(9.65, 10.13)
Smoking only	0.66	1.27	1.64	2.51	1.00	1.8
	(0.60, 0.73)	(1.17, 1.37)	(1.50, 1.78)	(2.35, 2.67)	(0.93, 1.07)	(1.70, 1.89)
Smokeless only	3.86	6.46	7.52	9.76	5.11	7.85
	(3.70, 4.02)	(6.22, 6.69)	(7.18, 7.87)	(9.43, 10.09)	(4.94, 5.28)	(7.64, 8.07)
Dual use	0.1	0.15	0.26	0.36	0.15	0.24
	(0.07, 0.12)	(0.13, 0.18)	(0.20, 0.31)	(0.31, 0.42)	(0.13, 0.18)	(0.21, 0.27)
Education						
No education	20.57	37.25	36.83	53.22	26.13	44.01
	(20.19, 20.95)	(36.76, 37.74)	(36.18, 37.49)	(52.63, 53.81)	(25.76, 26.50)	(43.60, 44.41)
Primary	12.44	19.03	14.24	17.97	13.06	18.58
	(12.15, 12.74)	(18.64, 19.41)	(13.79, 14.70)	(17.53, 18.41)	(12.80, 13.32)	(18.28, 18.88)
Secondary	54.32	41.25	41.82	27.37	50.05	35.38
	(53.81, 54.82)	(40.74, 41.76)	(41.12, 42.53)	(26.84, 27.91)	(49.61, 50.48)	(34.99, 35.76)
Higher	12.67	2.48	7.1	1.44	10.76	2.04
	(12.29, 13.05)	(2.26, 2.70)	(6.69, 7.50)	(1.28, 1.60)	(10.46, 11.07)	(1.89, 2.18)
Household size						
3 or less	12.35	9.02	12.9	15	12.54	11.55
	(12.00, 12.71)	(8.72, 9.32)	(12.44, 13.35)	(14.58, 15.42)	(12.25, 12.83)	(11.30, 11.81)
4 to 5	41.26	45.48	51.45	45.54	44.74	45.51
	(40.77, 41.74)	(44.99, 45.97)	(50.79, 52.11)	(44.96, 46.12)	(44.34, 45.15)	(45.12, 45.90)
6 to 8	30.4	31.48	27.73	31.15	29.49	31.34
	(29.94, 30.85)	(31.06, 31.91)	(27.19, 28.28)	(30.64, 31.65)	(29.13, 29.85)	(31.01, 31.68)
9 or more	15.99	14.01	7.92	8.31	13.23	11.6
	(15.61, 16.38)	(13.66, 14.37)	(7.60, 8.24)	(8.03, 8.60)	(12.94, 13.53)	(11.35, 11.86)
Wealth index quintiles						
1st (Poorest)	16.46	25.41	16.46	20.15	16.46	23.18
	(16.11, 16.82)	(24.95, 25.87)	(16.02, 16.89)	(19.71, 20.58)	(16.17, 16.75)	(22.82, 23.55)
2nd (Poorer)	18.59	24.63	17.62	22.17	18.26	23.59
	(18.22, 18.96)	(24.20, 25.06)	(17.15, 18.08)	(21.71, 22.63)	(17.95, 18.56)	(23.25, 23.93)
3rd (Middle)	20.99	22.54	18.83	22.08	20.25	22.35
	(20.57, 21.42)	(22.10, 22.98)	(18.30, 19.36)	(21.60, 22.57)	(19.91, 20.60)	(22.00, 22.69)
4th (Richer)	23.21	17.73	21.7	20.59	22.69	18.94
	(22.72, 23.69)	(17.29, 18.18)	(21.10, 22.29)	(20.07, 21.11)	(22.28, 23.10)	(18.58, 19.30)
5th (Richest)	20.75	9.69	25.4	15.01	22.34	11.94
	(20.22, 21.28)	(9.28, 10.09)	(24.70, 26.10)	(14.47, 15.55)	(21.87, 22.80)	(11.57, 12.31)
Religion						
Hindu	81.18	81.92	81.01	83.4	81.12	82.55
	(80.60, 81.76)	(81.32, 82.53)	(80.37, 81.64)	(82.83, 83.97)	(80.61, 81.63)	(82.03, 83.06)
Muslim	13.62	14.32	12.43	13.09	13.21	13.8
	(13.07, 14.16)	(13.76, 14.88)	(11.88, 12.99)	(12.57, 13.62)	(12.73, 13.69)	(13.32, 14.28)
Christian	1.8	1.51	2.37	1.43	1.99	1.48
	(1.64, 1.95)	(1.35, 1.66)	(2.12, 2.62)	(1.28, 1.59)	(1.84, 2.15)	(1.35, 1.60)
Sikh	1.8	0.69	2.64	0.71	2.09	0.69
	(1.70, 1.91)	(0.60, 0.77)	(2.45, 2.83)	(0.64, 0.78)	(1.98, 2.20)	(0.63, 0.76)
Buddhist	0.98	0.71	0.93	0.88	0.96	0.78
	(0.83, 1.12)	(0.59, 0.82)	(0.74, 1.12)	(0.70, 1.06)	(0.83, 1.09)	(0.67, 0.89)
Other	0.63	0.86	0.62	0.49	0.62	0.7
	(0.49, 0.76)	(0.65, 1.08)	(0.51, 0.73)	(0.40, 0.58)	(0.52, 0.72)	(0.55, 0.85)
Caste						
Not backward class	26.25	22.8	29.28	24.43	27.29	23.49
	(25.67, 26.84)	(22.23, 23.36)	(28.54, 30.01)	(23.81, 25.05)	(26.78, 27.80)	(23.00, 23.98)
Scheduled caste	20.01	22.75	18.14	21.99	19.37	22.43
	(19.48, 20.54)	(22.19, 23.31)	(17.58, 18.71)	(21.36, 22.63)	(18.93, 19.82)	(21.94, 22.92)
Scheduled tribe	8.83	10.9	8.06	8.97	8.57	10.09
	(8.52, 9.15)	(10.52, 11.28)	(7.70, 8.42)	(8.61, 9.34)	(8.30, 8.84)	(9.77, 10.40)
Other backward class	44.9	43.55	44.52	44.61	44.77	44
	(44.28, 45.52)	(42.95, 44.15)	(43.79, 45.25)	(43.94, 45.28)	(44.24, 45.30)	(43.48, 44.51)
Region						
Rural	66.42	74.59	29.53	70.47	65.08	72.85
	(65.76, 67.08)	(73.96, 75.22)	(28.86, 30.20)	(69.80, 71.14)	(64.56, 65.59)	(72.33, 73.37)
Urban	33.58	25.41	37.51	62.49	34.92	27.15
	(32.92, 34.24)	(24.78, 26.04)	(36.78, 38.25)	(61.75, 63.22)	(34.41, 35.44)	(26.63, 27.67)
Observations	97,905	92,646	51,969	67,433	149,874	160,079

95% confidence intervals are in parenthesis

Estimates are obtained using complex survey weights

Shares add to 100 across rows for respective characteristics

**Fig. 2:**
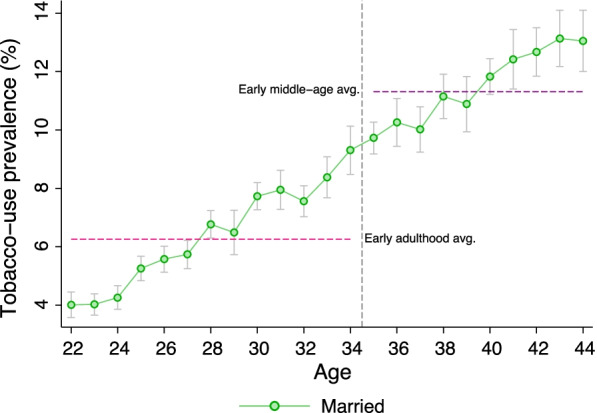
Tobacco use prevalence of early adult and early middle-aged women in India by age. Estimates were obtained using complex survey weights

**Fig. 3:**
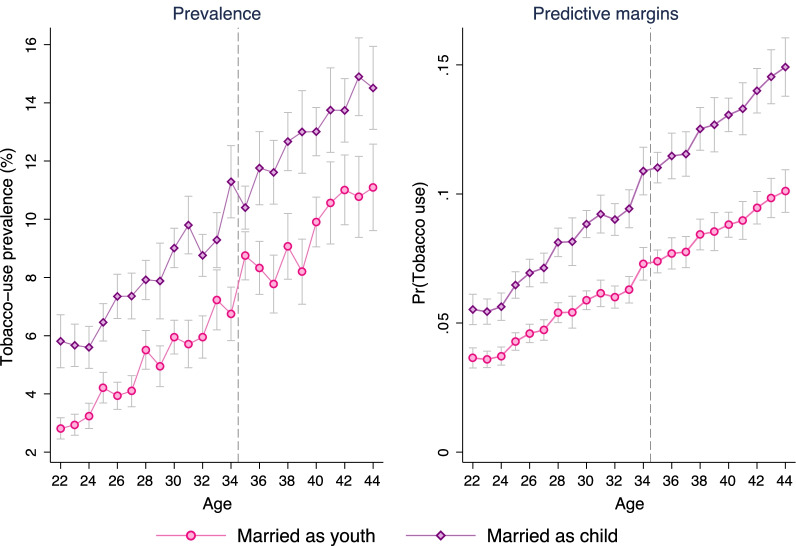
Difference in tobacco use prevalence and predictive margins by age and child marriage. Estimates were obtained using complex survey weights

Tobacco use prevalence was respectively 6% and 11% among early adult and early middle-aged women (Fig. 2). Around 80% of the tobacco users consumed smokeless tobacco only and nearly 2% consumed both smoking- and smokeless-tobacco products. For both early adult and early middle-aged groups, tobacco use prevalence was around 3%-points higher among those who were married before age 18 than their peers married as youth (18 to 21). The difference in tobacco-use prevalence were observed at every age level (Fig. 3). The differences were also observed across household wealth levels in both urban and rural areas (see Additional file 1: Appendix). The differences were statistically significant ($p < 0.10$) for all age and household wealth index categories except the rural poorest quintile for early middle-age women.

**Fig. 4:**
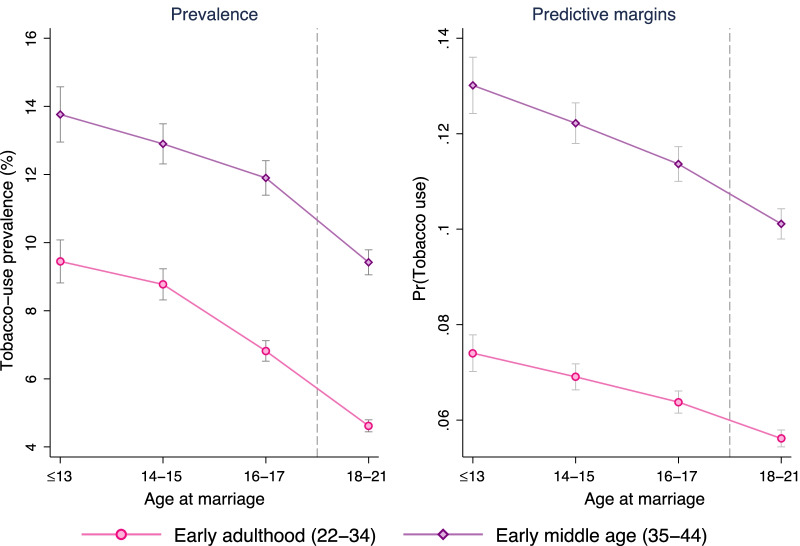
Tobacco use prevalence and predictive margin by early adulthood and early middle-age and age at marriage. Estimates were obtained using complex survey weights. Vertical lines across the markers represent 95% confidence intervals

Figure 4 illustrates the tobacco use prevalence and predictive margins by age at marriage categories for early adult and early middle-aged women. Prevalence for both groups not only varied by child marriage (before 18 years) but also by age at the time of child marriage—the lower the marital age, the higher the risk of tobacco use. At early adulthood, prevalence of tobacco use was approximately 9% among those who were married by age 13, while it was approximately 7% among those married during age 16 to 17 years. Similar trends were observed at early middle-age, in which prevalence was around 14% and 12% among those who were married by age 13 and during age 16 to 17, respectively. The predictive margins after controlling for the sociodemographic correlates, however, were relatively smaller than the actual prevalence rates. These prevalence patterns and predictive margins were similar in both urban and rural areas (see Additional file 1: Appendix).

Table 2 presents the odds ratio (OR) and adjusted odds ratio (AOR) in favor of tobacco use for child marriage and other covariates. In the univariate specification, the odds of tobacco use for those who were married as a child were 1.8 and 1.4 times that of those who were married as a youth among early adult and early middle-aged women, respectively. After controlling for other covariates in the multivariable specification, the AORs were 1.3 and 1.2 for the two groups, respectively. Age was significantly associated with tobacco use as the odds of tobacco use increased by 1.1 times for 1 year increase in age. Among other covariates, the odds of tobacco use were higher among women who were Muslim, resided in urban areas, and belonged to scheduled castes or tribes. Conversely the odds of tobacco use were lower among women from wealthier households, and with higher educational attainment.

**Table 2:** Odds ratios and adjusted odds ratios in favor of tobacco use for child marriage and other covariates

	Unadjusted			Adjusted		
	Early adulthood	Early middle age	All	Early adulthood	Early middle age	All
Child marriage	1.767***	1.390***	1.644***	1.265***	1.168***	1.222***
	(1.685, 1.853)	(1.322, 1.462)	(1.588, 1.702)	(1.200, 1.333)	(1.105, 1.235)	(1.177, 1.268)
Age				1.070***	1.045***	1.059***
				(1.061, 1.078)	(1.035, 1.054)	(1.055, 1.062)
Education						
No education				Ref.	Ref.	Ref.
Primary				0.797***	0.776***	0.793***
				(0.737, 0.862)	(0.719, 0.838)	(0.749, 0.841)
Secondary				0.450***	0.464***	0.450***
				(0.411, 0.492)	(0.425, 0.507)	(0.422, 0.481)
Higher				0.139***	0.208***	0.158***
				(0.098, 0.196)	(0.134, 0.322)	(0.121, 0.206)
Household size						
3 or less				Ref.	Ref.	Ref.
4 to 5				0.916*	0.994	0.970
				(0.832, 1.008)	(0.912, 1.082)	(0.912, 1.032)
6 to 8				0.867***	1.034	0.959
				(0.786, 0.956)	(0.946, 1.130)	(0.898, 1.023)
9 or more				0.900*	1.105	0.992
				(0.794, 1.019)	(0.974, 1.255)	(0.906, 1.086)
Wealth index quintiles						
1st (Poorest)				Ref.	Ref.	Ref.
2nd (Poorer)				0.683***	0.797***	0.733***
				(0.639, 0.730)	(0.746, 0.852)	(0.698, 0.769)
3rd (Middle)				0.515***	0.579***	0.542***
				(0.472, 0.562)	(0.535, 0.627)	(0.509, 0.576)
4th (Richer)				0.333***	0.443***	0.385***
				(0.294, 0.378)	(0.396, 0.496)	(0.353, 0.420)
5th (Richest)				0.167***	0.241***	0.205***
				(0.132, 0.212)	(0.203, 0.286)	(0.179, 0.236)
Religion						
Hindu				Ref.	Ref.	Ref.
Muslim				1.244***	1.330***	1.287***
				(1.109, 1.395)	(1.197, 1.477)	(1.184, 1.400)
Christian				0.703**	0.654***	0.677***
				(0.534, 0.926)	(0.529, 0.808)	(0.562, 0.815)
Sikh				0.054***	0.275***	0.181***
				(0.012, 0.238)	(0.131, 0.580)	(0.094, 0.349)
Buddhist				0.691*	0.807	0.756**
				(0.466, 1.024)	(0.553, 1.177)	(0.573, 0.998)
Other				1.017	1.274	1.108
				(0.768, 1.348)	(0.951, 1.708)	(0.889, 1.380)
Caste						
Not backward class				Ref.	Ref.	Ref.
Scheduled caste				1.320***	1.379***	1.348***
				(1.172, 1.487)	(1.237, 1.537)	(1.237, 1.469)
Scheduled tribe				1.798***	1.746***	1.779***
				(1.579, 2.048)	(1.548, 1.969)	(1.615, 1.960)
Other backward class				0.988	1.063	1.028
				(0.887, 1.101)	(0.968, 1.168)	(0.953, 1.108)
Residence						
Rural				Ref.	Ref.	Ref.
Urban				1.460***	1.299***	1.380***
				(1.308, 1.630)	(1.173, 1.439)	(1.269, 1.501)
Observations	190,551	119,402	309,953	190,069	119,281	309,681

95% confidence intervals are in parenthesis

***p < 0.01, **p < 0.05, *p < 0.1

Estimates are obtained using complex survey weights

All models control for state fixed effects. There were no tobacco-user early-adult women in the Chandigarh and Daman & Diu territories. There were no tobacco-user early-middle-aged women in the Chandigarh territory. Observations from these areas were omitted in the respective multivariable specifications

The relative risk ratios (RRR) and adjusted relative risk ratios (ARRR) in favor of mutually exclusive types of tobacco use for child marriage is presented in Table 3. Among the early adult women, the relative risk of smoking-, smokeless- and dual-tobacco use for the child brides were respectively 2.0, 1.7 and 1.6 times that of those who were married at or after age 18. Among the early middle-aged women, the RRRs were 1.6, 1.3, and 1.5 respectively. The adjusted relative risks were similar though lesser in magnitude and not statistically significant for the dual-use outcome.

**Table 3:** Relative risk ratios in favor of mutually exclusive types of tobacco use for child marriage

	Tobacco-use type			
	No tobacco	Smoking only	Smokeless only	Dual use
**Panel A. Early adult—Unadjusted**				
Child marriage	Base outcome	1.980***	1.733***	1.645***
		(1.766, 2.219)	(1.644, 1.828)	(1.231, 2.198)
**Panel B. Early adult—Adjusted**				
Child marriage	Base outcome	1.434***	1.238***	1.135
		(1.270, 1.619)	(1.167, 1.312)	(0.828, 1.556)
**Panel C. Early middle-age—Unadjusted**				
Child marriage	Base outcome	1.587***	1.345***	1.459***
		(1.434, 1.755)	(1.270, 1.424)	(1.133, 1.879)
**Panel D. Early middle-age—Adjusted**				
Child marriage	Base outcome	1.247***	1.147***	1.223
		(1.121, 1.387)	(1.078, 1.221)	(0.928, 1.612)
**Panel E. All—Unadjusted**				
Child marriage	Base	1.871***	1.599***	1.660***
		(1.733, 2.020)	(1.538, 1.662)	(1.372, 2.009)
**Panel F. All—Adjusted**				
Child marriage	Base	1.331***	1.199***	1.189
		(1.228, 1.443)	(1.149, 1.250)	(0.963, 1.470)

95% confidence intervals are in parenthesis

***p < 0.01

Estimates are obtained using complex survey weights

Each panel (A, B, C, D, E, and F) presents results of separate regressions

In the “other covariates” specification the following covariates (not reported here) were controlled for: age, education, household size, religion, caste, and state fixed effect

Following the findings that child marriage was associated with tobacco consumption in early adulthood and early middle-age, we then assessed the relationship at the intensive margin (i.e., by age when child marriage was occurred). We evaluated whether women who experienced child marriage at an earlier age were more likely to consume tobacco compared to women who experienced child marriage at a later age. We present the ORs and AORs for marital age and other covariates in Table 4. Among the women married as children, the younger the marital age, the greater was the risk of tobacco use as an adult. Among early adult women, the odds in favor of tobacco use for those who were married by age 13 years were 2.2 times that of those who were married as youth (18 to 21). For those who were married during age 14 to 15 and during age 16 to 17, the odds were respectively 2.0 and 1.5 times that of those who were married during age 18 to 21. After controlling for other covariates, the adjusted odds for those who were married by age 13, during age 14 to 15, and age 16 to 17 were respectively 1.4, 1.3, and 1.2 times that of those who were married during age 18 to 21. This pattern of higher odds of tobacco use for child marriage at a younger age was similarly observed among early middle-aged women. The odds among child marriage age categories (13, age 14 to 15, and age 16 to 17) were respectively 1.5, 1.4, and 1.3 times that of marriage as a youth. The adjusted odds were a bit smaller—1.3, 1.2, and 1.1 respectively, when other covariates were controlled for.

**Table 4:** Odds ratios and adjusted odds ratios in favor of tobacco use for age at marriage and other covariates

	Unadjusted			Adjusted		
	Early adulthood	Early middle age	All	Early adulthood	Early middle age	All
Age at marriage						
18 to 21	Ref.	Ref.	Ref.	Ref.	Ref.	Ref.
13 or less	2.155***	1.534***	1.944***	1.414***	1.293***	1.359***
	(1.987, 2.336)	(1.417, 1.662)	(1.833, 2.062)	(1.292, 1.547)	(1.185, 1.411)	(1.273, 1.451)
14 to 15	1.986***	1.424***	1.775***	1.332***	1.168***	1.252***
	(1.859, 2.122)	(1.335, 1.519)	(1.696, 1.857)	(1.237, 1.434)	(1.087, 1.256)	(1.191, 1.317)
16 to 17	1.511***	1.298***	1.446***	1.170***	1.113***	1.146***
	(1.429, 1.597)	(1.223, 1.378)	(1.388, 1.506)	(1.103, 1.242)	(1.044, 1.187)	(1.097, 1.197)
Age				1.069***	1.045***	1.059***
				(1.061, 1.077)	(1.036, 1.055)	(1.055, 1.062)
Education						
No education				Ref.	Ref.	Ref.
Primary				0.801***	0.778***	0.796***
				(0.740, 0.867)	(0.721, 0.840)	(0.751, 0.844)
Secondary				0.456***	0.469***	0.456***
				(0.417, 0.499)	(0.429, 0.513)	(0.427, 0.487)
Higher				0.140***	0.210***	0.160***
				(0.099, 0.198)	(0.135, 0.326)	(0.122, 0.209)
Household size						
3 or less				Ref.	Ref.	Ref.
4 to 5				0.916*	0.998	0.973
				(0.833, 1.008)	(0.916, 1.087)	(0.915, 1.036)
6 to 8				0.866***	1.037	0.961
				(0.785, 0.955)	(0.948, 1.133)	(0.900, 1.025)
9 or more				0.901	1.105	0.993
				(0.796, 1.021)	(0.973, 1.254)	(0.907, 1.088)
Wealth index quintiles						
1st (Poorest)				Ref.	Ref.	Ref.
2nd (Poorer)				0.683***	0.796***	0.732***
				(0.639, 0.730)	(0.745, 0.851)	(0.698, 0.769)
3rd (Middle)				0.516***	0.578***	0.541***
				(0.473, 0.563)	(0.534, 0.626)	(0.509, 0.575)
4th (Richer)				0.334***	0.443***	0.385***
				(0.295, 0.378)	(0.396, 0.495)	(0.352, 0.420)
5th (Richest)				0.168***	0.241***	0.205***
				(0.132, 0.213)	(0.203, 0.285)	(0.179, 0.235)
Religion						
Hindu				Ref.	Ref.	Ref.
Muslim				1.244***	1.328***	1.287***
				(1.109, 1.396)	(1.196, 1.476)	(1.184, 1.399)
Christian				0.706**	0.655***	0.679***
				(0.536, 0.930)	(0.530, 0.810)	(0.564, 0.817)
Sikh				0.054***	0.276***	0.181***
				(0.012, 0.238)	(0.131, 0.580)	(0.094, 0.349)
Buddhist				0.691*	0.808	0.756**
				(0.466, 1.026)	(0.555, 1.178)	(0.573, 0.998)
Other				1.025	1.284*	1.117
				(0.772, 1.359)	(0.958, 1.723)	(0.895, 1.393)
Caste						
Not backward class				Ref.	Ref.	Ref.
Scheduled caste				1.320***	1.377***	1.347***
				(1.171, 1.488)	(1.235, 1.535)	(1.236, 1.469)
Scheduled tribe				1.800***	1.746***	1.781***
				(1.580, 2.051)	(1.547, 1.969)	(1.616, 1.963)
Other backward class				0.990	1.065	1.030
				(0.888, 1.102)	(0.970, 1.170)	(0.955, 1.111)
Residence						
Rural				Ref.	Ref.	Ref.
Urban				1.461***	1.300***	1.381***
				(1.309, 1.631)	(1.174, 1.440)	(1.270, 1.503)
Observations	190,551	119,402	309,953	190,069	119,281	309,681

95% confidence intervals are in parenthesis

***p < 0.01, **p < 0.05, *p < 0.1

Estimates are obtained using complex survey weights

All models control for state fixed effects. There were no tobacco-user early-adult women in the Chandigarh and Daman & Diu territories. There were no tobacco-user early-middle-aged women in the Chandigarh territory. Observations from these areas were omitted in the respective multivariate specifications

Lastly, Table 5 shows the odds and adjusted odds in favor of tobacco use for child marriage by sub-sample of women by household wealth (poor/non-poor) and urban/rural residence. The odds and adjusted odds of tobacco use for those who were married as child were higher than that of those who were married at youth for all socioeconomic and educational sub-groups. The low (primary or no education) educated child brides were 1.3 times and 1.1 times more likely to consume tobacco in early adulthood and early middle-age respectively, than their low educated peers married during age 18 to 21. Similar was the finding for women with high (secondary or higher) educational attainment - child brides were 1.6 and 1.4 times more likely to consume tobacco at respective age categories. For other socioeconomic categories, the odds were the highest among the“urban non-poor” sub-group and the lowest among the “rural poor” subgroup for both early adulthood and early middle-age groups. For the early adult group, this pattern persisted when other covariates were controlled for. For the early middle-age group, the adjusted odds, however, were the highest for the “urban poor” sub-group and not statistically significant for the “urban non-poor” sub-group.

**Table 5:** Odds ratios and adjusted odds ratios in favor of tobacco use for child marriage by urban and rural household wealth and education sub-groups

	Unadjusted			Adjusted		
	Early adulthood	Early middle age	All	Early adulthood	Early middle age	All
**Panel A. Urban-poor**						
Child marriage	1.530***	1.383***	1.527***	1.191**	1.246**	1.231***
	(1.325, 1.766)	(1.170, 1.636)	(1.381, 1.689)	(1.018, 1.392)	(1.030, 1.507)	(1.104, 1.373)
Observations	23,403	13,953	37,356	23,258	13,923	37,306
**Panel B. Urban-nonpoor**						
Child marriage	2.509***	1.667***	2.141***	1.865***	1.116	1.335***
	(1.950, 3.229)	(1.294, 2.147)	(1.764, 2.599)	(1.425, 2.441)	(0.859, 1.449)	(1.078, 1.654)
Observations	23,639	18,877	42,516	21,671	17,675	42,043
**Panel C. Rural-poor**						
Child marriage	1.265***	1.071*	1.197***	1.215***	1.120***	1.167***
	(1.187, 1.347)	(1.000, 1.148)	(1.142, 1.255)	(1.136, 1.300)	(1.040, 1.207)	(1.110, 1.227)
Observations	59,587	33,808	93,395	59,542	33,806	93,395
**Panel D. Rural-nonpoor**						
Child marriage	1.744***	1.365***	1.669***	1.338***	1.163***	1.253***
	(1.608, 1.892)	(1.260, 1.479)	(1.575, 1.768)	(1.224, 1.464)	(1.065, 1.270)	(1.175, 1.335)
Observations	83,922	52,764	136,686	80,667	52,624	136,433
**Panel E. Low education**						
Child marriage	1.252***	1.095***	1.182***	1.242***	1.117***	1.173***
	(1.182, 1.326)	(1.035, 1.160)	(1.134, 1.232)	(1.168, 1.321)	(1.050, 1.189)	(1.123, 1.226)
Observations	89,173	77,383	166,556	89,021	77,334	166,447
**Panel F. High education**						
Child marriage	1.626***	1.373***	1.559***	1.396***	1.213***	1.326***
	(1.488, 1.776)	(1.220, 1.544)	(1.452, 1.674)	(1.265, 1.540)	(1.064, 1.382)	(1.225, 1.436)
Observations	101,378	42,019	143,397	98,408	39,156	138,624

95% confidence intervals are in parenthesis

***p < 0.01, **p < 0.05, *p < 0.1

Estimates are obtained using complex survey weights

Each panel (A, B, C, D, E, and F) presents results of separate regressions

Poor refers to households in the bottom-two (1st and 2nd) wealth index quintiles of respective regions (urban or rural). Non-poor refers to households in the top-three (3rd, 4th, and 5th) wealth index quintiles of respective regions (urban or rural)

Low education refers to no education or primary education. High education refers to secondary or higher education

In multivariable specification (adjusted) the following covariates (not reported here) were controlled for: age, education, household size, religion, caste, and state fixed effect

Of the 28 states, the odds in favor of tobacco-use were higher ($p < 0.10$) for those who were married as child than their peers in 22 and 17 states respectively for the early adult and early middle-age groups. While the adjusted odds were also higher ($p < 0.10$) in majority (17) of the states for the early adult group, they were higher ($p < 0.10$) in 6 states for the early middle-age group. The OR for the early adult group was the highest in Andhra Pradesh, followed by Punjab, Goa, Uttarakhand, and Tamil Nadu. For the early middle-age group, the OR was the highest in Kerala, followed by Uttarakhand, Tamil Nadu, Telangana, and Haryana (see Additional file 1: Appendix). These estimates of sub-group analysis render the robustness of our general findings.

## Discussion

The relationship between child marriage, a childhood experience with damaging health implications, and tobacco product use in young and early middle-aged adult females is less visited in extant literature. We contribute to the literature by examining this relationship in a LMIC. Results indicate that after controlling for relevant covariates, women married as children were more likely to consume tobacco products in early adulthood and early middle-age compared to women who were married as youths. Our findings are consistent with previous studies on the associations between tobacco use and adverse childhood experiences both in developed [19, 34, 35] and developing [36, 37] country settings. Importantly, the extent of child marriage in the intensive margin (i.e., age when child marriage occurred) matters. Among women married as children, younger marital age was associated with higher risk of adult tobacco use. While examining the heterogeneity in the association between tobacco use and child marriage across different geographic (i.e., urban/ rural) and wealth groups, the risk was found the greatest among “urban non-poor” sub-group and the lowest among the “rural poor” sub-group for both early adult and early middle-age groups. Across states, the risk was observed higher in several southern states including Andhra Pradesh, Tamil Nadu, Telangana, and Karnataka. However, irrespective of the geographic and wealth groupings, the risks of tobacco use in adulthood were significantly greater among child brides compared to their peers.

There are a number of mechanistic channels that may explain the observed linkage between these two important public health concerns. Extant work suggests that tobacco use is a strategy to relieve negative social and emotional sequelae following exposure to childhood adversity [38]. In other words, tobacco could be an avoidant coping technique against child-marriage-related depression [7] as well as notable behavioral outcomes such as lack of sexual autonomy and low bargaining power in patriarchal households [8]. Child marriage may also dampen access to support systems promoting resilience for young women, such as school, friends, and employment [5], which may affect their mental health and in turn lead to tobacco consumption. Moreover, a young bride may be more malleable compared to an adult and thus pick up tobacco use from the husband’s household.

Additional evidence suggests that young women with a history of child abuse are at twice the risk for early smoking initiation compared to their non-abused peers (Jun et al. 2008; Anda et al. 1999). While this study did not examine the prevalence of “abuse experiences” among women married as children, child marriage heightens the risk for intimate partner violence [39], which plausibly compounds physical and psychological harm on this vulnerable group. Another argument of increasing attention is the potential interplay between genetic and environmental factors on smoking risk, where epigenetic changes following early life stress exposure increases nicotine dependence—however, the only study to date suggests a sex-dependent relationship particularly among males [40].

Tobacco control is relevant to at least six of the seventeen United Nations Sustainable Development Goals (SDGs); and directly related to the SDG target of reducing premature deaths from noncommunicable diseases (NCDs) by one third by 2030 [41]. Consuming chewing tobacco is a significant risk factor of oral cancer among women in India [42]. Tobacco user women in India are also more likely to have uncontrolled hypertension [43]. Reducing tobacco use, therefore, may lower the population level risks of cancer and cardiovascular diseases, and associated mortality. To this end, the finding that child marriage is associated with tobacco consumption uncovers one potential lever—preventing child marriage to decrease tobacco consumption. Thus, we offer a link between the SDG targets of eliminating child marriage (target 5.3) and reducing premature mortality from NCDs (target 3.4). Coordinated interventions will augment public health efforts to achieve these two SDG targets especially in countries like India where both child marriage and tobacco use are critical public health concerns.

### Limitations

The study is subject to several limitations. Due to data constraints, we could not examine the mental health conditions to validate the psychosocial factors that may be driving the tobacco use among women who were married as a child. Because of the cross-sectional nature of data, and the lack of any policy variation in terms of minimum age at marriage, we could not offer any causal relationship between child marriage and tobacco use. We do not know whether tobacco use was initiated before or after marriage, nor do we know if a former-user quitted tobacco at some point and became a tobacco non-user at the time of the survey. It may also be the case that girls who are more likely to be tobacco consumers are also more likely to be married as a child because of their socioeconomic conditions. To mitigate these issues we analyzed various socioeconomic sub-samples of relatively lower degree of heterogeneity across respondents. Nevertheless, our results suggest a persisting relationship between child marriage and tobacco use in adulthood, which has important public health implications.

## Conclusions

Our findings generate evidence on the association between child marriage and tobacco use as a behavioral health risk over the life course. Policy implications are twofold. First, enforcing nationwide bans on child marriage may simultaneously strengthen tobacco control and decrease the risk of downstream health problems. Second, anti-tobacco policies or cessation programs should explore ways of targeting child brides, who disproportionately bear the burden of tobacco-use compared to their peers. Further research is needed to develop and evaluate tailored sociocultural programs against child marriage and tobacco-use, especially in regions where risk may be greatest.

## Supplementary Information

**Additional file 1**. Additional analyses without marital age restrictions, and by urban and rural wealth index quintiles and state of residence.

## Data Availability

The datasets used and/or analyzed during the current study are freely available from the USAID’s Demographic and Health Surveys (DHS) Program website (https://www.dhsprogram.com/data/dataset_admin/login_main.cfm) upon registering as a DHS data user and submitting a research proposal.

## Abbreviations

ACE: Adverse childhood experience
LMIC: Low-and-middle income country
NFHS-4: India National Family Health Survey Round 4
SDG: Sustainable development goals
NCD: Noncommunicable diseases
