# Supplementary material for: Child marriage and risky health behaviors: an analysis of tobacco use among early adult and early middle-aged women in India
Source: BMC Womens Health. 2022 Jun 3;22:206. doi: 10.1186/s12905-022-01781-3 (PMC9164419; doi:10.1186/s12905-022-01781-3)
Supplement: Supplementary file 1 — Additional file 1. Additional analyses without marital age restrictions, and by urban and rural wealth index quintiles and state of residence. [file 12905_2022_1781_MOESM1_ESM.pdf]

A Appendix

Figure A.1: **Difference in tobacco use prevalence of women aged 22 to 44 in India across urban and rural household wealth index quintiles by child marriage.** Estimates were obtained using complex survey weights. Vertical lines across the markers represent 95% confidence intervals.

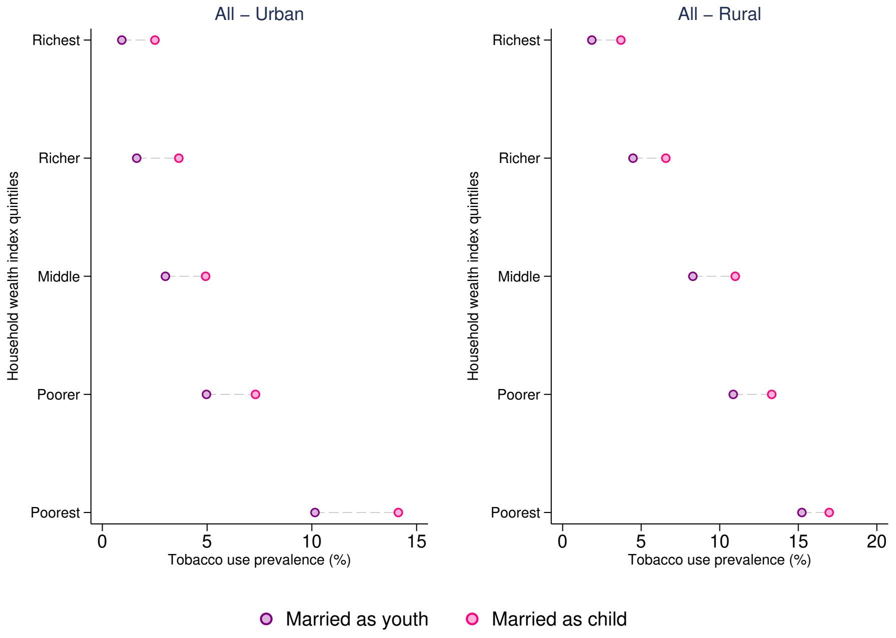

Figure A.2: **Difference in tobacco use prevalence of early-adult women in India across urban and rural household wealth index quintiles by child marriage.** Estimates were obtained using complex survey weights. Vertical lines across the markers represent 95% confidence intervals.

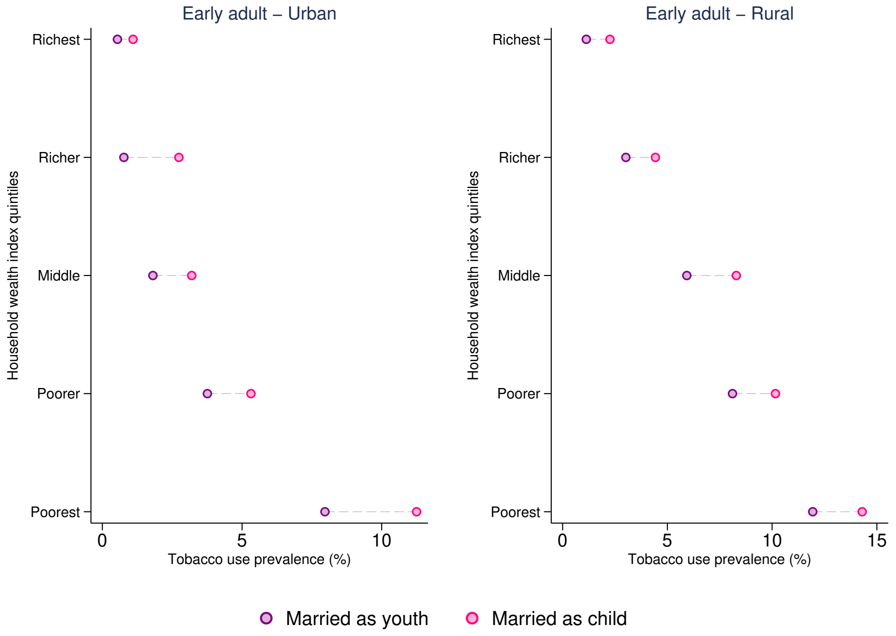

Figure A.3: **Difference in tobacco use prevalence of early-middle-aged women in India across urban and rural household wealth index quintiles by child marriage.** Estimates were obtained using complex survey weights. Vertical lines across the markers represent 95% confidence intervals.

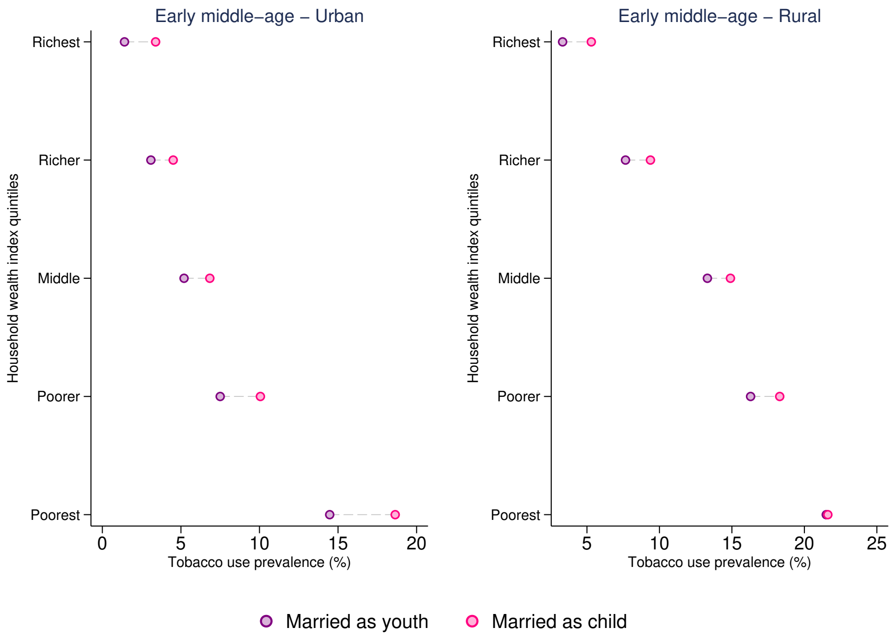

Figure A.4: **Tobacco use prevalence and predictive margin by early adulthood and early middle-age and age at marriage in rural areas.** Estimates were obtained using complex survey weights. Vertical lines across the markers represent 95% confidence intervals.

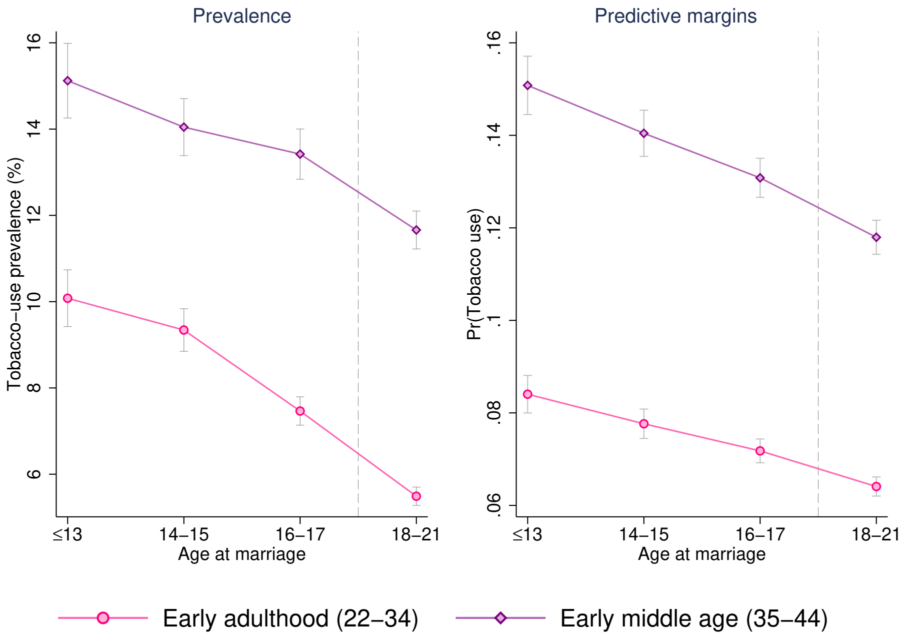

Figure A.5: **Tobacco use prevalence and predictive margin by early adulthood and early middle-age and age at marriage in urban areas.** Estimates were obtained using complex survey weights. Vertical lines across the markers represent 95% confidence intervals.

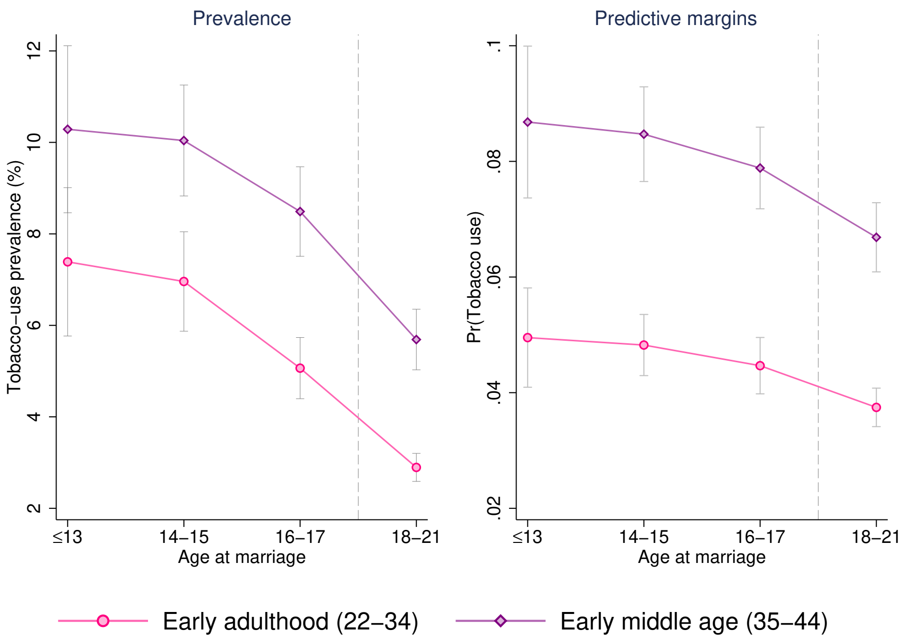

Table A.1: Odds ratios and adjusted odds ratios in favor of tobacco use for child marriage and other covariates - Age 18 to 49 - no restriction on marital age

	Univariate	Multivariable
Child marriage	2.055***	1.281***
	(1.997, 2.114)	(1.241, 1.321)
Age		1.057***
		(1.055, 1.059)
Education		
No education		Ref.
Primary		0.796***
		(0.759, 0.834)
Secondary		0.420***
		(0.399, 0.442)
Higher		0.166***
		(0.143, 0.192)
Household size		
3 or less		Ref.
4 to 5		0.981
		(0.938, 1.027)
6 to 8		0.979
		(0.935, 1.027)
9 or more		1.055
		(0.978, 1.137)
Wealth index quintiles		
1st (Poorest)		Ref.
2nd (Poorer)		0.747***
		(0.717, 0.777)
3rd (Middle)		0.564***
		(0.536, 0.593)
4th (Richer)		0.403***
		(0.376, 0.431)
5th (Richest)		0.233***
		(0.209, 0.259)
Religion		
Hindu		Ref.
Muslim		1.303***
		(1.213, 1.400)
Christian		0.674***
		(0.590, 0.769)
Sikh		0.209***
		(0.115, 0.380)
Buddhist		0.805*
		(0.633, 1.023)
Other		1.170*
		(0.999, 1.370)
Caste		
Not backward class		Ref.
Scheduled caste		1.373***
		(1.281, 1.472)
Scheduled tribe		1.799***
		(1.659, 1.951)
Other backward class		1.029
		(0.968, 1.094)
Residence		
Rural		Ref.
Urban		1.318***
		(1.227, 1.414)
Constant		
Observations	596,418	596,418

95% confidence intervals are in parenthesis.
$***p < 0.01$, $**p < 0.05$, $*p < 0.1$.
Estimates are obtained using complex survey weights.
All models control for state fixed effects.

Table A.2: Relative risk ratios in favor of mutually exclusive types of tobacco use for child marriage - Age 18 to 49 - no restriction on marital age

	Tobacco-use type			
	No tobacco	Smoking only	Smokeless only	Dual use
**Panel A. Age 18-49 – No other covariates**				
Child marriage	Base	2.295***	1.998***	2.309***
		(2.157, 2.443)	(1.936, 2.062)	(2.009, 2.654)
**Panel B. Age 18-49 – Other covariates**				
Child marriage	Base	1.292***	1.273***	1.390***
		(1.212, 1.376)	(1.229, 1.318)	(1.183, 1.633)

95% confidence intervals are in parenthesis.
$***p < 0.01$, $**p < 0.05$, $*p < 0.1$.
Estimates are obtained using complex survey weights.
Each panel (A and B) presents results of separate regressions.
In the "other covariates" specification the following covariates (not reported here) were controlled for: age, education, household size, religion, caste, and state fixed effect.

Table A.3: Odds ratios and adjusted odds ratios in favor of tobacco use for age at marriage and other covariates - Age 18 to 49 - no restriction on marital age

	Univariate	Multivariable
Age at marriage		
18 to 21		Ref.
13 or less	2.529***	1.439***
	(2.404, 2.662)	(1.359, 1.524)
14 to 15	2.257***	1.309***
	(2.173, 2.345)	(1.255, 1.366)
16 to 17	1.762***	1.200***
	(1.701, 1.824)	(1.155, 1.245)
Age		1.057***
		(1.055, 1.059)
Education		
No education		Ref.
Primary		0.798***
		(0.762, 0.836)
Secondary		0.424***
		(0.403, 0.447)
Higher		0.166***
		(0.144, 0.193)
Household size		
3 or less		Ref.
4 to 5		0.983
		(0.939, 1.029)
6 to 8		0.98
		(0.935, 1.027)
9 or more		1.054
		(0.978, 1.137)
Wealth index quintiles		
1st (Poorest)		Ref.
2nd (Poorer)		0.746***
		(0.717, 0.777)
3rd (Middle)		0.564***
		(0.536, 0.593)
4th (Richer)		0.402***
		(0.376, 0.430)
5th (Richest)		0.232***
		(0.209, 0.259)
Religion		
Hindu		Ref.
Muslim		1.302***
		(1.212, 1.399)
Christian		0.675***
		(0.591, 0.770)
Sikh		0.209***
		(0.115, 0.380)
Buddhist		0.805*
		(0.634, 1.022)
Other		1.176**
		(1.003, 1.377)
Caste	.	
Not backward class		Ref.
Scheduled caste		1.372***
		(1.280, 1.471)
Scheduled tribe		1.801***
		(1.661, 1.953)
Other backward class		1.031
		(0.969, 1.096)
Region		
Rural		Ref.
Urban		1.318***
		(1.228, 1.415)
Observations	596,408	596,408

95% confidence intervals are in parenthesis.
$***p < 0.01$, $**p < 0.05$, $*p < 0.1$.
Estimates are obtained using complex survey weights.
All models control for state fixed effects.

Table A.4: Odds ratios and adjusted odds ratios in favor of tobacco use for child marriage and other covariates - no marital age restriction

	Univariate			Multivariable		
	Early Adulthood	Early Middle age	All	Early Adulthood	Early Middle age	All
Child marriage	2.109***	1.555***	1.937***	1.297***	1.214***	1.262***
	(2.016, 2.206)	(1.488, 1.626)	(1.875, 2.001)	(1.235, 1.363)	(1.155, 1.276)	(1.218, 1.308)
Age				1.069***	1.044***	1.059***
				(1.061, 1.076)	(1.036, 1.053)	(1.056, 1.062)
Education						
No education				Ref.	Ref.	Ref.
Primary				0.792***	0.780***	0.794***
				(0.736, 0.851)	(0.726, 0.839)	(0.752, 0.838)
Secondary				0.426***	0.466***	0.438***
				(0.393, 0.462)	(0.429, 0.505)	(0.413, 0.465)
Higher				0.162***	0.193***	0.169***
				(0.135, 0.196)	(0.150, 0.248)	(0.143, 0.199)
Household size						
3 or less				Ref.	Ref.	Ref.
4 to 5				0.900**	0.991	0.956
				(0.827, 0.980)	(0.918, 1.070)	(0.904, 1.012)
6 to 8				0.854***	1.03	0.944*
				(0.782, 0.933)	(0.950, 1.116)	(0.891, 1.001)
9 or more				0.918	1.087	0.993
				(0.822, 1.025)	(0.966, 1.223)	(0.913, 1.079)
Wealth index quintiles						
1st (Poorest)				Ref.	Ref.	Ref.
2nd (Poorer)				0.674***	0.808***	0.732***
				(0.633, 0.717)	(0.760, 0.860)	(0.700, 0.766)
3rd (Middle)				0.511***	0.589***	0.544***
				(0.472, 0.554)	(0.547, 0.634)	(0.514, 0.576)
4th (Richer)				0.329***	0.445***	0.383***
				(0.295, 0.368)	(0.401, 0.494)	(0.354, 0.414)
5th (Richest)				0.183***	0.243***	0.212***
				(0.150, 0.224)	(0.209, 0.283)	(0.186, 0.241)
Religion						
Hindu				Ref.	Ref.	Ref.
Muslim				1.277***	1.335***	1.303***
				(1.146, 1.423)	(1.211, 1.472)	(1.201, 1.414)
Christian				0.730***	0.622***	0.674***
				(0.595, 0.895)	(0.520, 0.744)	(0.582, 0.782)
Sikh				0.130***	0.229***	0.191***
				(0.031, 0.550)	(0.115, 0.459)	(0.101, 0.361)
Buddhist				0.732*	0.821	0.778*
				(0.507, 1.056)	(0.584, 1.153)	(0.602, 1.005)
Other				1.059	1.324*	1.153
				(0.839, 1.337)	(0.991, 1.768)	(0.946, 1.404)
Caste						
Not backward class				Ref.	Ref.	Ref.
Scheduled caste				1.350***	1.387***	1.362***
				(1.214, 1.502)	(1.255, 1.533)	(1.257, 1.475)
Scheduled tribe				1.787***	1.740***	1.765***
				(1.599, 1.997)	(1.553, 1.949)	(1.614, 1.929)
Other backward class				0.995	1.07	1.032
				(0.905, 1.095)	(0.982, 1.167)	(0.962, 1.106)
Residence						
Rural						
Urban				1.478***	1.268***	1.374***
				(1.334, 1.637)	(1.154, 1.393)	(1.270, 1.486)
Observations	278,362	155,412	433,774	277,467	155,412	433,774

95% confidence intervals are in parenthesis.
$***p < 0.01$, $**p < 0.05$, $*p < 0.1$.
Estimates are obtained using complex survey weights.
All models control for state fixed effects.

Table A.5: Relative risk ratios in favor of mutually exclusive types of tobacco use for child marriage - no marital age restriction

	Tobacco-use type			
	No tobacco	Smoking only	Smokeless only	Dual use
**Panel A. Early adult – No other covariates**				
Child marriage	Base	2.276***	2.082***	1.959***
		(2.037, 2.543)	(1.983, 2.187)	(1.528, 2.512)
**Panel B. Early adult – Other covariates**				
Child marriage	Base	1.410***	1.277***	1.221
		(1.264, 1.573)	(1.208, 1.350)	(0.922, 1.617)
**Panel C. Early middle-age – No other covariates**				
Child marriage	Base	1.848***	1.490***	1.693***
		(1.685, 2.028)	(1.418, 1.566)	(1.355, 2.113)
**Panel D. Early middle-age – Other covariates**				
Child marriage	Base	1.261***	1.199***	1.289**
		(1.142, 1.392)	(1.135, 1.268)	(1.000, 1.661)
**Panel E. All – No other covariates**				
Child marriage	Base	2.219***	1.880***	1.999***
		(2.063, 2.387)	(1.813, 1.949)	(1.697, 2.355)
**Panel F. All – Other covariates**				
Child marriage	Base	1.322***	1.248***	1.254**
		(1.227, 1.423)	(1.198, 1.299)	(1.031, 1.525)

95% confidence intervals are in parenthesis.

$***p < 0.01$, $**p < 0.05$, $*p < 0.1$.

Estimates are obtained using complex survey weights.

Each panel (A, B, C, D, E, and F) presents results of separate regressions.

In the "other covariates" specification the following covariates (not reported here) were controlled for: age, education, household size, religion, caste, and state fixed effect.

Table A.6: Odds ratios and adjusted odds ratios in favor of tobacco use for age at marriage and other covariates - no martial age restriction

	Univariate			Multivariable		
	Early Adulthood	Early Middle age	All	Early Adulthood	Early Middle age	All
Age at marriage						
18 to 21	2.571***	1.717***	2.290***	1.444***	1.342***	1.400***
13 or less	(2.375, 2.784)	(1.592, 1.851)	(2.162, 2.426)	(1.322, 1.577)	(1.235, 1.458)	(1.312, 1.493)
	2.370***	1.593***	2.091***	1.364***	1.212***	1.292***
14 to 15	(2.227, 2.522)	(1.500, 1.692)	(2.002, 2.184)	(1.272, 1.462)	(1.133, 1.297)	(1.232, 1.355)
	1.803***	1.453***	1.703***	1.203***	1.158***	1.186***
16 to 17	(1.706, 1.905)	(1.375, 1.535)	(1.637, 1.772)	(1.135, 1.275)	(1.090, 1.229)	(1.137, 1.238)
Age				1.068***	1.044***	1.059***
				(1.061, 1.076)	(1.036, 1.053)	(1.055, 1.062)
Education						
No education						
Primary				0.795***	0.782***	0.796***
				(0.740, 0.855)	(0.728, 0.841)	(0.754, 0.841)
Secondary				0.431***	0.470***	0.443***
				(0.397, 0.467)	(0.433, 0.510)	(0.417, 0.470)
Higher				0.164***	0.194***	0.170***
				(0.136, 0.197)	(0.151, 0.250)	(0.144, 0.200)
Household size						
3 or less				0.901**	0.994	0.959
4 to 5				(0.827, 0.981)	(0.921, 1.074)	(0.906, 1.015)
				0.854***	1.032	0.946*
6 to 8				(0.782, 0.932)	(0.952, 1.119)	(0.892, 1.002)
				0.919	1.086	0.994
9 or more				(0.823, 1.027)	(0.965, 1.223)	(0.914, 1.080)
Wealth index quintiles						
1st (Poorest)				Ref.	Ref.	Ref.
2nd (Poorer)				0.674***	0.808***	0.732***
				(0.633, 0.717)	(0.759, 0.859)	(0.700, 0.766)
3rd (Middle)				0.512***	0.588***	0.544***
				(0.473, 0.554)	(0.546, 0.633)	(0.514, 0.576)
4th (Richer)				0.330***	0.444***	0.382***
				(0.295, 0.368)	(0.400, 0.493)	(0.353, 0.413)
5th (Richest)				0.184***	0.242***	0.212***
				(0.150, 0.225)	(0.208, 0.282)	(0.186, 0.240)
Religion						
Hindu				Ref.	Ref.	Ref.
Muslim				1.277***	1.334***	1.303***
				(1.146, 1.423)	(1.210, 1.471)	(1.201, 1.413)
Christian				0.731***	0.623***	0.676***
				(0.596, 0.897)	(0.521, 0.745)	(0.583, 0.783)
Sikh				0.130***	0.230***	0.191***
				(0.031, 0.550)	(0.115, 0.459)	(0.101, 0.361)
Buddhist				0.733*	0.822	0.778*
				(0.508, 1.056)	(0.585, 1.154)	(0.603, 1.004)
Other				1.065	1.332*	1.16
				(0.843, 1.346)	(0.997, 1.780)	(0.951, 1.415)
Caste						
Not backward class				Ref.	Ref.	Ref.
Scheduled caste				1.350***	1.385***	1.361***
				(1.213, 1.502)	(1.253, 1.531)	(1.256, 1.474)
Scheduled tribe				1.789***	1.740***	1.766***
				(1.600, 2.000)	(1.553, 1.949)	(1.615, 1.931)
Other backward class				0.996	1.072	1.033
				(0.905, 1.096)	(0.983, 1.169)	(0.963, 1.108)
Residence						
Rural				Ref.	Ref.	Ref.
Urban				1.479***	1.268***	1.374***
				(1.335, 1.639)	(1.154, 1.393)	(1.270, 1.487)
Observations	278,356	155,409	433,765	277,461	155,409	433,765

95% confidence intervals are in parenthesis.
$***p < 0.01$, $**p < 0.05$, $*p < 0.1$.
Estimates are obtained using complex survey weights.
All models control for state fixed effects.

Table A.7: Odds ratios and adjusted odds ratios in favor of tobacco use for child marriage by state

	Univariate			Multivariable		
	Early Adulthood	Early Middle age	All	Early Adulthood	Early Middle age	All
Andhra Pradesh	5.901***	0.992	2.095***	4.176***	0.773	1.365
	(2.344, 14.856)	(0.580, 1.697)	(1.341, 3.275)	(1.644, 10.611)	(0.444, 1.348)	(0.848, 2.195)
Arunachal Pradesh	1.422***	0.959	1.183**	1.334***	1.089	1.197**
	(1.156, 1.750)	(0.773, 1.190)	(1.015, 1.377)	(1.081, 1.646)	(0.871, 1.360)	(1.026, 1.397)
Assam	1.268***	1.163**	1.240***	1.1	1.119	1.112**
	(1.111, 1.448)	(1.001, 1.351)	(1.124, 1.369)	(0.955, 1.266)	(0.959, 1.306)	(1.003, 1.231)
Bihar	1.525***	1.245*	1.441***	1.271*	1.196	1.221**
	(1.153, 2.018)	(0.980, 1.580)	(1.206, 1.721)	(0.959, 1.685)	(0.939, 1.522)	(1.019, 1.462)
Chhattisgarh	1.834***	1.370***	1.770***	1.258***	1.115	1.211***
	(1.611, 2.088)	(1.168, 1.608)	(1.601, 1.957)	(1.092, 1.449)	(0.942, 1.320)	(1.088, 1.348)
Goa	3.741*	1.547	2.031	7.997**	0.846	1.611
	(0.804, 17.404)	(0.378, 6.325)	(0.693, 5.952)	(1.092, 58.566)	(0.168, 4.270)	(0.495, 5.248)
Gujarat	2.062***	1.513***	1.801***	1.227	1.109	1.179*
	(1.630, 2.607)	(1.151, 1.989)	(1.511, 2.146)	(0.961, 1.567)	(0.837, 1.471)	(0.980, 1.419)
Haryana	2.692**	1.938***	2.346***	1.307	1.470*	1.477**
	(1.239, 5.848)	(1.249, 3.008)	(1.595, 3.451)	(0.590, 2.893)	(0.936, 2.309)	(1.002, 2.178)
Himachal Pradesh	1.609	1.717	1.965	0.929	0.972	0.768
	(0.255, 10.172)	(0.578, 5.102)	(0.776, 4.973)	(0.132, 6.566)	(0.369, 2.558)	(0.332, 1.776)
Jharkhand	1.587***	0.942	1.234***	1.342**	0.914	1.113
	(1.266, 1.988)	(0.779, 1.138)	(1.068, 1.426)	(1.060, 1.699)	(0.752, 1.112)	(0.960, 1.291)
Karnataka	2.501***	1.773***	2.207***	1.895***	1.32	1.527***
	(1.827, 3.425)	(1.312, 2.395)	(1.831, 2.660)	(1.398, 2.569)	(0.944, 1.844)	(1.195, 1.951)
Kerala	0.835	2.847**	1.757*	0.374*	1.643	0.871
	(0.374, 1.866)	(1.219, 6.648)	(0.939, 3.288)	(0.124, 1.127)	(0.545, 4.957)	(0.453, 1.675)
Madhya Pradesh	1.481***	1.288***	1.460***	1.051	1.113	1.083*
	(1.336, 1.640)	(1.130, 1.469)	(1.347, 1.583)	(0.942, 1.173)	(0.972, 1.274)	(0.994, 1.180)
Maharashtra	2.182***	1.374**	1.831***	1.329**	1.059	1.187*
	(1.688, 2.821)	(1.062, 1.778)	(1.538, 2.181)	(1.009, 1.750)	(0.803, 1.396)	(0.994, 1.416)
Manipur	1.524***	0.93	1.280***	1.330**	0.924	1.165
	(1.223, 1.899)	(0.706, 1.224)	(1.070, 1.531)	(1.058, 1.671)	(0.694, 1.231)	(0.969, 1.400)
Meghalaya	1.154	1.207	1.172*	0.977	1.159	1.035
	(0.910, 1.463)	(0.917, 1.590)	(0.980, 1.401)	(0.765, 1.247)	(0.857, 1.568)	(0.861, 1.243)
Mizoram	0.977	1.163	1.036	0.942	1.274	1.03
	(0.676, 1.410)	(0.823, 1.643)	(0.803, 1.337)	(0.661, 1.344)	(0.913, 1.776)	(0.809, 1.312)
Nagaland	1.195	0.979	1.1	1.213*	0.941	1.094
	(0.960, 1.488)	(0.755, 1.269)	(0.928, 1.304)	(0.974, 1.512)	(0.707, 1.252)	(0.916, 1.306)
Odisha	1.840***	1.331***	1.664***	1.333***	1.149*	1.247***
	(1.603, 2.113)	(1.162, 1.526)	(1.508, 1.836)	(1.148, 1.547)	(0.994, 1.328)	(1.122, 1.386)
Punjab	4.272**	2.159	2.474	2.837*	1.284	1.0000
	(1.360, 13.418)	(0.526, 8.862)	(0.830, 7.371)	(0.932, 8.630)	(0.196, 8.435)	(0.322, 3.100)
Rajasthan	1.606***	1.209**	1.441***	1.213**	1.114	1.169**
	(1.370, 1.883)	(1.013, 1.443)	(1.281, 1.622)	(1.025, 1.437)	(0.926, 1.339)	(1.036, 1.319)
Sikkim	1.273	1.228	1.266	1.096	1.152	1.078
	(0.813, 1.995)	(0.720, 2.095)	(0.893, 1.797)	(0.698, 1.721)	(0.668, 1.988)	(0.762, 1.525)
Tamil Nadu	3.134***	2.179***	2.768***	1.958***	1.490**	1.651***
	(1.948, 5.042)	(1.580, 3.007)	(2.130, 3.597)	(1.176, 3.260)	(1.051, 2.112)	(1.254, 2.174)
Tripura	1.671***	1.374*	1.578***	1.330**	1.125	1.260**
	(1.282, 2.177)	(0.996, 1.897)	(1.274, 1.955)	(1.001, 1.767)	(0.781, 1.621)	(1.003, 1.583)
Uttar Pradesh	1.961***	1.454***	1.823***	1.436***	1.320***	1.377***
	(1.763, 2.180)	(1.314, 1.609)	(1.693, 1.963)	(1.283, 1.606)	(1.191, 1.464)	(1.276, 1.487)
Uttarakhand	3.290***	2.227***	2.942***	1.897**	1.437*	1.572***
	(1.958, 5.529)	(1.557, 3.187)	(2.191, 3.951)	(1.107, 3.252)	(0.980, 2.106)	(1.144, 2.160)
West Bengal	1.658***	1.296**	1.476***	1.134	1.226*	1.213**
	(1.322, 2.080)	(1.051, 1.597)	(1.260, 1.728)	(0.904, 1.422)	(0.985, 1.527)	(1.034, 1.423)
Telangana	2.290*	1.975*	2.505***	1.191	1.4	1.274
	(0.951, 5.512)	(0.956, 4.081)	(1.422, 4.414)	(0.543, 2.612)	(0.639, 3.067)	(0.747, 2.174)

95% confidence intervals are in parenthesis.

$***p < 0.01$, $**p < 0.05$, $*p < 0.1$.

Estimates are obtained using complex survey weights.

Regressions were separately estimated for each state.

In multivariate specification the following covariates (not reported here) were controlled for: age, education, household size, religion, and caste.
